# The Compassionate Communities Connectors programme: experiences of supported families and referring healthcare providers

**DOI:** 10.1177/26323524231173705

**Published:** 2023-05-12

**Authors:** Samar M Aoun, John Rosenberg, Robyn Richmond, Bruce Rumbold

**Affiliations:** The University of Western Australia, Perth, WA 6009, Australia; Perron Institute for Neurological and Translational Science, Perth, WA 6009, Australia; La Trobe University, Melbourne, VIC 3086, Australia; University of the Sunshine Coast, Sunshine Coast, QLD, Australia; Perron Institute for Neurological and Translational Science, Perth, WA, Australia; La Trobe University, Melbourne, VIC, Australia; Perron Institute for Neurological and Translational Science, Perth, WA, Australia

**Keywords:** Caring Helpers, chronic disease, community networks, Compassionate Communities, Connectors, end-of-life care, palliative care, social connectedness, social isolation, volunteers

## Abstract

**Background and Aim:**

Comprehensive evaluations that include the experience of patients and service providers are vital if interventions are to be translated into the standard practice of health services and allow formal networks to work as partners with informal community networks. However, published evaluations are limited in the palliative care volunteering literature. The objective of the study is to explore the experiences and views of both patients and their family carers who received support and their referring healthcare providers concerning their participation in the Compassionate Communities Connectors programme, in the south-west region of Western Australia. Connectors identified and addressed gaps in community and healthcare provision by accessing resources and mobilising social networks of people with life-limiting illnesses. The perspectives of patients, carers and service providers concerning the feasibility and acceptability of the intervention were sought.

**Methods:**

Semistructured interviews were undertaken with 28 patients/families and 12 healthcare providers, resulting in 47 interviews in total (March 2021-April 2022). An inductive content analysis was used in analysing interview transcripts to identify key themes.

**Results:**

Families greatly appreciated the support and enablement received from the Connectors. Healthcare providers were impressed with the high level of resourcefulness exhibited by the Connectors and perceived a great need for the programme, particularly for those socially isolated. Three themes captured the patients’/families’ perspectives: connector as an advocate, increasing social connectedness and taking the pressure off families. Healthcare providers’ perspectives were captured in three themes: reducing social isolation, filling a gap in service provision and building the capacity of the service.

**Conclusions:**

Perspectives of patients/families and healthcare providers demonstrated the mediating role of Connectors. Each group saw the Connectors’ contribution through the lens of their particular interests or needs. However, there were indications that the connection was shifting the way each group understood and practised care, encouraging or restoring agency to families and reminding healthcare providers that collaborating beyond the boundaries of their roles actually enhances the whole ecology of care. Using a Compassionate Communities approach to mobilise health and community sectors has the potential to develop a more holistic approach that addresses the social, practical and emotional domains of care.

## Introduction

Volunteers have been an integral part of palliative care and hospice services since the late 1970s,^1^ playing an essential role in providing emotional and practical care for dying people and their families.^1–3^ Increasingly, volunteers have an expanded role through building social networks and community-development activities.^4–11^

Palliative care volunteers are considered valuable contributors to the multidisciplinary team.^12^ Volunteers are rated as valuable by healthcare staff for being compassionate, caring and good listeners, with 75% of nurses in one study reporting that having volunteers involved made their jobs easier.^13^ Volunteers are also viewed as informal or symbolic links between the hospice and the community,^5,6,14^ creating links between the institution and the general public by ‘bringing the world in’ and ‘taking the hospice out into the community’.^6^ However, another study reports that only 43% of hospital nurses and 63% of community nurses thought that volunteers should have a role in linking the healthcare team and the family.^13^

Family caregivers also value the role palliative care volunteers play in physical and emotional care.^7^ Williams *et al.*^15^ interviewed family caregivers about their experiences as ‘navigators’ for their family members. Navigators provided enabling support and information that can assist patients in accessing treatments or services that support their wellbeing. Family members reported several barriers to acting as a navigator for their family members, including lack of clarity about the role and reluctance to add yet another person to their care team. These barriers, and the ongoing need to actively advocate for their family, were often described as distressing for the family and created additional stress for caregivers. They also noted healthcare providers could discredit the knowledge of family caregivers. Williams *et al.*^15^ argue that there is a need for programmes and services that support family caregivers in navigating these gaps in systems and processes between informal and formal caregiving. Rosenberg *et al.*^16^ reiterate this, noting that ‘service providers were most helpful when they recognised the informal caring network and facilitated good communication, sharing of expertise and relationship building between formal and informal carers’ (p. 8).

Patients, family members and healthcare teams may also benefit from programmes that reduce social isolation by enhancing existing social networks,^17–21^ reinforcing the connection with everyday life and creating a sense of normality for patients and their families.^22^ Furthermore, mobilising support provides a way to legitimise asking for help, which can transform the caring experience for carers, caring networks and health professionals.^22–24^ Sallnow^25^ notes that compassionate community-based volunteering that recruited from existing social networks had an impact on the way the hospice interacted with volunteers. In particular, recruitment practices changed to be more inclusive of people who spoke English as a second language because volunteers now reflected the communities in which patients lived. This in turn resulted in more equitable access for both volunteers and community members interacting with the hospice.

Public health approaches to end-of-life care invite review of the roles and contributions of all participants, including volunteers. However, there is still little research and evaluation in the palliative care volunteering literature on the feedback from patients/families and service providers that could inform health services wishing to take such initiatives into their standard practice. This article adds the consumers’ and providers’ perspectives to the findings of the Compassionate Communities Connectors programme previously described and reported.^11,26–28^

### Description of the programme

The programme is a volunteer-led initiative designed to enhance the social networks of families living with chronic or life-limiting illnesses. It is an initiative of the South West Compassionate Communities Network in Western Australia, in partnership with regional health services. Palliative care, chronic disease and aged care health service teams refer families to the programme, and the project coordinator matches these families with Connectors to ensure the fit is as good as possible. The development, implementation and evaluation of the programme were extensively described previously,^28^ demonstrating significant improvements in social connectedness.

Specially trained volunteers support the social networks of existing members of the families and enlist the support of community members, Caring Helpers, to address the social and practical needs of these families. Connectors attend a 2-day training course delivered by content experts. Sessions include information on public health palliative care, the importance of compassionate communities and how this project fits in, death literacy and advance care planning, grief literacy, communication skills, self-care, understanding advanced illness and the role of palliative care and chronic disease teams and volunteer processes and procedures within the health service.^28^

This article reports on interviews with patients/family members and their referring healthcare providers concerning their experiences of the programme. A previous article has reported and discussed feedback from the Connectors.^27^ A final article will report the impact of the programme on service utilisation.

## Objective

The objective of this study was to explore the experiences and views of patients/family carers who received support and their referring healthcare providers concerning their participation in the Compassionate Communities Connectors programme. Their perspectives of the feasibility and acceptability of the intervention were particularly sought.

This particular aspect of the study draws on an evaluation undertaken at the end of the initial pilot study period. Results already are informing the continued implementation of the programme now incorporated in standard practice of regional health services.

## Methods

Ethics approval (RGS3419) was obtained from the WA Country Health Service Human Research Ethics Committee and La Trobe University Ethics Committee. All participants signed a consent form to participate and were able to withdraw their consent at any time.

In this community connector programme, implementation is shaped by the actual circumstances encountered and the feedback of participants. It has been conducted using a participatory action method approach. The programme design has evolved through the contribution of Connectors, participating health professionals and the research team. This is a classic action method that draws upon the reflexivity of all involved, and it is inherent in implementing the programme and informs the way the evaluation findings are understood.

### Participants

Forty-three patients/family carers participated in the trial and were supported by 13 Connectors. The average number of families supported by each connector during the study period was three, with a range between one and nine families. Families were followed up by Connectors for a median of 18 weeks, ranging from 3 to 52 weeks.^28^

#### Patient profile

Sixty-three percent of patients were female, with a median age of 76 years (range 36-90 years); 57% were living alone; 40% had cancer as their primary diagnosis, followed by cardiac/respiratory (34%); 47% were married and 53% were widowed, divorced, separated or single and 97% were retired or on a disability pension.^28^

#### Healthcare provider profile

Healthcare professionals came from palliative care teams, chronic disease teams and aged care teams of the health service. They were nurses and social workers and all female except for one male. No other demographic information was collected.

### Data collection

Semistructured interviews were undertaken with 28 patients/families and 12 healthcare providers (47 interviews in total as some healthcare providers were interviewed more than once regarding different patients; Table 1). Interviews took place from March 2021 to April 2022. Interviews were audio-recorded and transcribed verbatim. All identifying information was removed.

**Table 1.** Completed interviews by the two target groups.

Participant Group	Number of Interviews	Duration in min (range)	Mean (SD)
Patient/family carer	28	4.53-26.28	9.55 (0.27)
Healthcare provider	19	2.30-21.18	6.44 (0.17)

SD, standard deviation.

Apart from one face-to-face interview, all interviews were conducted by telephone. Interviews took place after patients/families had completed their period of intervention.

#### Interview protocols

The brief interview protocol comprised broad questions to the families such as: Please describe your experience with the connector and what ways has the connector helped you. What has been less than positive (difficult) about accepting help from Connectors? Do you have any suggestions that would make having a compassionate connector more beneficial? What help have you got from the Caring Helpers? What has been less than positive (difficult) about accepting help from Caring Helpers? What would you share with others about having a Compassionate connector? What would you share with others about accessing support from Caring Helpers? Is there anything else you would like to share?

The brief interview protocol with the referring healthcare providers comprised the following questions: What was your experience with the Compassionate Connector project? What has been less than positive about the Compassionate Connector project? What, if anything, has changed about your practice since being involved in the Compassionate Connector project? Is there anything else you would like to share?

### Data analysis

In this qualitative study, inductive content analysis was utilised in reviewing audio-recordings and transcripts of the interviews. This was undertaken by the interviewer and other members of the research team, generating open codes and enabling identification of emergent themes. Interviews in effect gathered up the informal learning that has taken place throughout the implementation of the project. Furthermore, feedback on the preliminary findings was sought from a participating volunteer and a healthcare provider. These analytical steps supported verification of the findings from multiple sources.

## Results

Overall, six themes were identified (Table 2); three from the experience of the patients/family carers and three from the healthcare providers’ experience.

**Table 2.** Themes from feedback by the two target groups.

Patients/families
Connector as an advocate – having someone at your back
Increasing social connectedness – opening up their world
Taking the pressure off families
Healthcare providers
Reducing social isolation – life changing
Filling a gap in service provision – more layers of support
Building the capacity of the service – another string to the bow

### Feedback from patients/family carers

#### Theme one: connector as an advocate–having someone at your back

Patients/families felt a strong connection with their Connectors and recognised them as people they could trust and who would advocate for them:

You’ve got someone at your back, if you like . . . if you need anything. If I really feel I need something extra, I know I can ring [Connector]. To find out the information that I’d like. (ID31)

This advocacy by the Connector supported patients/families to feel more enabled and confident to speak up for themselves with both formal and informal service providers:

[Connector] had said to me, if the people that come out to see you to help you are not suitable . . ., it’s okay to say, “Can you send someone different?” And I did. I actually rang. And I said, you know, “Is it possible that we can have someone that is maybe older?” maybe has some connections, because I could see the shine in my husband’s eyes. And that’s what I wanted to see, when I get home. And they said, “Yes, you can do that. That’s fine. Thanks for telling us”. And I thought, oh, [Connector] told me I can do this and it’s alright to do it. So yes, in that way, [Connector] connected both of us socially. (ID78)

Families saw Connectors as genuine people who were there because they wanted to be there; their role as a neutral voice assisted them to advocate for families:

It wasn’t that I didn’t have family support. I had absolutely. And you know, for me, sometimes stepping out of the family is quite good. Yeah. It’s a bit neutral. With someone neutral you’re not always guarded in what you say. (ID78)

She sounds really genuine, you know? And then if she says, oh, I’ll get in touch and do this next thing. I know she’s done it. (ID88)

It was evident that Connectors were highly valued by families who described them as helpful, reliable, supportive and friendly. Families also described how much they looked forward to their visits, knowing that the connector had the families’ interests at heart:

Oh, look, I would say just looking forward to her visits, her pleasant attitude, and her willing to do things for us if we needed it. (ID54)

She wasn’t there to tell me what to do. She was there to help me make sense of what was going on in my life. (ID13)

Some patients resisted accepting a connector into their lives as it took time for them to understand this role, but eventually they felt the benefits:

[Connector] was wonderful because I didn’t really want her to come the first couple of times. I wasn’t very nice to her. Well, I wasn’t very encouraging let’s put that way, but she won me over. So give her a gold star’. (ID108)

#### Theme two: increasing social connectedness – opening up their world

Families talked about the role that Connectors had in reducing their isolation and improving their social connections. One family member described it in this way:

If you can imagine a fully blown up football . . . if that was my life, it now feels like a golf ball. I can see my golf ball is sort of growing a tiny little bit more maybe to a tennis ball size, but oh, that’s great. (ID123)

Connectors were reported as providing social encounters to patients/families; arranging one-on-one visits with the Caring Helpers; identifying potential external social groups/events; people to walk pets; meal trains and arranging transport for shopping/appointments:

Yeah, it is communication. We can talk about things and it’s nice to be able to talk about what you used to do and all that type of thing. And she also communicates very well with [husband] as well. So, it’s all about communication. It opens you up and you don’t feel quite so isolated. (ID31)

It is a testament to the quality of the Connectors and Caring Helpers how these interactions have resulted in continuing friendships:

They’ve become like friends, you know, they feel the same way, they enjoy doing it because we make a lovely friend. One said that to me the other day. (ID35)

Well [Caring Helper] comes and sees me on [day] and we have a laugh together. She’s such a funny lady, she really is. Caring and, oh, she’s lovely. And she’s helped me out with lots of different things, you know? She’s a real good friend now. She’s not just an acquaintance, she’s wonderful. (ID115)

One family carer spoke of the benefit in having someone external come in and encourage their patient to reduce their social isolation:

Well, when she first came, she talked [patient] into going to the Men’s Shed, which was really good cause I’d been trying to get him to go there for three years and refused to, and she came along and she said to him, you know, why don’t you go to the Men’s Shed? So he ended up going and he absolutely loved it. (ID10)

Knowing that someone was going to visit also provided a level of accountability from the patients:

One of the first things which was a catalyst for change was there’s a level of accountability if somebody’s coming to see you. You’ve got to get up, get dressed and you’ve got to face the world. You can’t just lay curled up in your bed all day. (ID13)

For partners who are full-time carers, this programme has allowed them the opportunity to get out and maintain their own social connectedness:

I think it’s still open up another area that if you are like myself can only go out when there’s somebody here looking after [husband]. Yeah. It opens you up to the outside world. (ID31)

#### Theme three: taking the pressure off families

Patients with a partner/family carer described how the additional support from a connector reduced the burden on their families and allowed the focus to shift from ‘caring jobs’ to enjoying their family time:

It takes the pressure off my immediate family, because they don’t have to take me to these things and I can call on the volunteers, which means that I’m not having to call on my family. . . . it’s a difficult time for them as well as me. (ID35)

I can go out and have a coffee with my sister and it’s, it’s not work-related. It’s just, we are going out for coffee because they cover the work-related things . . . (ID51)

Other patients who live on their own talked about the relief of having someone to help them do things that family normally would have done:

If you’re alone and don’t have any family, I’ve got no family in this country, they’re all overseas. And unfortunately, they can’t come because of this COVID. So [Connector] has been really, really helpful. She sort of done things that I would’ve asked my family to sort out. (ID49)

With the connector, they could be honest about how they were feeling, whereas often patients/family carers were unwilling to place additional burdens on their family. They felt comfortable talking with the Connectors and valued their discretion:

I did feel I could talk about anything and that she wouldn’t sort of tell people she would only tell them what I wanted other people to know . . . the word I’m looking for is discreet. Yeah, she was. And I think that is a big thing. Because sometimes you don’t want your business all over the town, you know? (ID49)

### Feedback from healthcare providers

#### Theme one: reducing social isolation – life changing

Service providers who referred patients/families to the Connectors’ programme acknowledged its positive impact on recipients:

I’ve got one patient in particular, yeah, it was, it was life changing. (HP021)

This was especially the case for those patients/families experiencing social isolation or even living alone; specifically, the impact of the Connectors’ programme upon mental health was lauded:

I think it’s a really worthwhile program, especially for those people who are struggling because they’re socially isolated and their mental health has then deteriorated from that. So what I’ve noticed is that people who once they feel like they’ve got a bit more social, a bit more of a social network, it seems to improve their mood and it just improves their capacity for self-care. (HP028)

Service providers overall reported they observed an increase in social connection and a reduction in social isolation for participants living alone with chronic and life-limiting health conditions. Social connections were viewed as important for the wellbeing of patients:

I think it’s a great service that’s actually helping very socially isolated people realize that there are people out there that care about them, and just knowing that there are people who care can help a lot, especially if there’s any mental health issues and things, but the social isolation side of things. So I think it’s terrific. I hope it continues. I really do so seriously. Just about every one of my clients could use one. (HP046)

#### Theme two: filling a gap in service provision – more layers of support

The complex nature of patient-family experiences was recognised by service providers as requiring a broader response than formal services could provide. When rural locations and the limits of families’ capacity to provide care were considered, these respondents stated,

I think it’s really bridged the gap, it goes beyond what a formal service can do and then it fills that gap of what family not able to do. So it’s that real. And I think they [Connectors] are a little bit more engaging. They are able to do a little bit more. It’s a good thing. (HP017)

I think the good side of it is that people in like smaller rural places because some services are not available here. So we have a lot of people who have to go to main city to see a palliative care specialist or PET scan, you know, stuff that they can’t actually do down in a smaller town. (HP003)

The Connectors’ programme was seen, then, as a valued response to limited formal services. Rather than an alternative, it was seen as complementary to services in addressing complexities of support:

It’s just great to be able to offer another layer of support because, as we know, it’s all a mine field, the whole cancer journey or dying process. So that’s great to be able to offer another service in, in a sort of a rural setting because whatever way you look at it, you do get less, sort of input than if you’re in a Metro [urban area]. So for even smaller places, long, further away from where their oncologist is, it’s certainly gotta be positive, offer more layers of support, isn’t it? (HP003)

Although this support was not clinical, it did enable extra observation of the impacts of this patient’s illness:

But [Connector] did help out sort of pinpoint problems with [patient]’s health condition, which we were then able to do something about, you know, like there were certain things [patient] wasn’t telling us about, her eating or her appetite . . . but there definitely is an extra pair of eyes on the ground for people like [patient] was very helpful. (HP070)

Interestingly, for patients/families who did not qualify for specific formal services (such as home-based aged care), the Connectors’ programme provided an option for referrers to ensure social support was available after discharge from hospital:

. . . they don’t actually qualify for formal services. So yeah, it’s a great, it’s a great thing. (HP055)

Service providers also reflected on differences between the roles of service providers and community Connectors, recognising the value of peer support over professional support:

For patients who are socially isolated, even just having the friendly contact of someone else in the community. And probably the fact that they aren’t particularly a health professional is good as well. Because they may, like a lot of our patients, have a lot of appointments and medical appointments where the power shift is probably felt like we have the power and they’re just doing whatever. I think the Connectors obviously do that differently and it is on more of a peer friendship level to be perceived. And I think that’s really helpful because we don’t get that anywhere else. And I think that has helped with engagement, just people being a little more proactive about themselves and being guided and pointed in the right direction. Rather than being quite so expectant of everything being fixed for them. (HP074)

#### Theme three: building the capacity of the service – another string to the bow

Service providers viewed the connector programme as enhancing the care provided by their health care service and was seen to build the capacity of their service and of individual healthcare professionals:

It’s just another string to the bow. (HP001)

I definitely love to have it as a tool in belt to offer more isolated people. (HP021)

Indeed, this healthcare professional described their promotion of these informal connections as a desirable element of communities, where such networks were generated according to need:

I guess what it would maybe encourage me to facilitate more people using those informal networks. I mean, I did that anyway, but I think seeing the positive impact that it has had to really encourage people either to use that project, but also to just do that themselves, to reach out . . . To reach out to those neighbours, to really engage in community. Whereas I think sometimes it’s, let’s just put the formal services in and we, because we are so busy, we just tick boxes. (HP017)

Furthermore, this respondent explicitly explains how hospital-based nurses could facilitate the mobilisation of social networks before discharge, if the programme was better embraced by them:

But you need to talk about it with the girls [nurses] that are actually on the floor talking to the patients and having those when you go home, you could try this and try this. They get in there really at that ground level with the patient. And I don’t think there’s a lot of nurses that really understand what the project’s all about. And sometimes from discharge home from hospital, like if they can start plant things, the seed in hospital, that would be really good . . . I know what an integral role those nurses play in influencing the patients about how they feel about going home and stuff. (HP021)

To achieve this, though, requires an ongoing effort. This participant expressed the view that reiteration of initial information would enable a more robust engagement by other healthcare professionals to better promote the programme for families in need:

I actually even think another information session with the palliative nurses as well. I don’t think we sell it hard enough. (HP021)

It is important to note that service providers felt that the programme reminded them about the importance of social connections and the social dimensions of healthcare. Ultimately, the value of the Connectors’ programme was clear in promoting a joint effort between formal services and social networks, in a range of contexts:

I think sometimes when you’re doing a formal service, you kind of feel like you have to have the answer where you have to find the right service. It certainly encouraged me to encourage people, and empower them to find their own, their own resources and their own connections. (HP037)

I think it’s just really reinforced for me the importance of social connections. (HP028)

## Discussion

These responses are part of an evaluation of the Compassionate Communities Connectors programme, a public health approach through which families, living with a person dying from advanced illness, are helped to reclaim and develop social capital and supportive connections within their community. The Connectors identified gaps in informal support and in community and health service provision, addressing these by accessing resources and mobilising networks as previously reported.^27,28^ Connectors are volunteers with the Compassionate Communities Connectors programme, but in contrast with most service-based volunteers, they have more agency in the way they can operate: Their role is not defined by the health services who refer to the programme. As explained earlier, the focus of this article is upon the experiences of participating family members and referring health professionals. The experience of Connectors and the impact of the programme on service utilisation are the subject of other complementary articles.^27,28^

Feedback from families in this study is similar to that reported by Pesut *et al.*^29^ who described many benefits about the strong relationship they built with Connectors. Families were grateful to have someone that they could turn to outside the family who was knowledgeable and supportive, reducing the burden on the extended family and providing a safety net. Participants were very appreciative of the opportunities for relationship building, having their autonomy respected and regaining their control over their situation. Contributing to these positive outcomes were the attributes of the Connectors, variously described as caring, friendly, nonintrusive, good listeners, trustworthy, uplifting, discreet and reliable.

However, it took time for families to understand the focus and nature of the Connectors’ role, initially showing reluctance to accept them, instead expecting more instrumental support from them. This was also reported by Pesut *et al.*^29^ Families were often unwilling to take up offers of Caring Helpers, as they wanted ‘their’ connector to remain as their contact point. This was also partly due to the often-overwhelming number of services and supports that come with being diagnosed with a chronic, life-limiting illness. The downside was that patients/families were often unwilling to transfer their expectations of practical support from Connectors to Caring Helpers, resulting in increasing workloads for Connectors as they took on more patients/families while also maintaining frequent connections with previous patients/families. This study showed that Connectors sourced about 60% of help through externally facilitated networks rather than naturally occurring networks, perhaps a consequence of about half of the patients living alone^28^ and being referred precisely because they had limited social networks. As the programme continues to develop, the focus needs to stay on Connectors enhancing people’s past and current social networks by facilitating others to join or return, rather than joining these networks themselves.

Our previous article reported a significant increase in the social connectedness of the participating patients and that 80% of the needs were found to be in the social domain.^28^ Looking at how Connectors and caring helpers improved social connectedness, it was more about the ‘little things’: ‘sharing a cuppa’ with them and chatting, walking the dog, gardening, assisting with transport and shopping. Pfaff *et al.*^30^ reported that these little things ‘had the biggest impact on client well-being and care delivery’ (p. 1). The little things were addressed by these authors through processes such as taking time, advocacy and empowerment.

Healthcare providers were impressed with the high level of resourcefulness that the Connectors exhibited, perceived a great need for the programme and understood that no formal service can address the complete needs of these families, particularly those living in some isolated, rural regions. They recognised that their time was insufficient to offer the type of care and extended conversations that the Connectors provided, and in some cases, they described these constraints on their time as reducing their input to just ‘ticking boxes’.

However, healthcare providers reported that participation in the programme increased the capacity of their service to provide good care and that their own practice was enhanced when they started encouraging their clients to ask their social networks for support. That is, participating in the programme made healthcare providers more aware of the resources and connections embedded in their patients’ social networks and encouraged the providers to develop and exercise skills in community advocacy. The healthcare providers also reported that patients or families were often more willing to open up to Connectors and that the frequency of visits meant that Connectors often had better oversight of how a patient/family was travelling than the service provider, hence the description of Connectors as ‘like an extra pair of eyes on the ground’ for the service providers.

Some of the responses of health care professionals imply that they saw Connectors as assisting them in their work, describing them as ‘just another string to their bow’ or ‘like an extra pair of eyes on the ground’ rather than seeing the Connectors as complementing and enhancing the professionals’ contribution. A clear implication of our findings, however, is that increasing the capacity of the service depends on healthcare providers recognising that Connectors provide something different, not just an extension of the health service. This recognition requires the service to engage differently with the community, to accept power-sharing and acknowledge that community members can make contributions that professionals cannot.

In terms of the community engagement model put forward by Sallnow and Paul,^31^ the Compassionate Communities Connectors programme is a cooperative project that has the potential to develop collaboratively into care that is coproduced by health and community services and the communities they serve. The responses reported here and in our earlier article on connector responses demonstrate attitudinal shifts that will support increased community engagement in the future. Connectors are becoming aware of their agency as members of their local community as they initiate supportive action; family members are becoming aware of the resources available to them in their own social networks and local communities as well as government-funded services and healthcare providers are becoming aware of the complementary contributions of community networks, especially as mediated through programmes like Compassionate Communities Connectors.

In suggesting ways to improve the programme, healthcare providers wanted to see more focused communication strategies to raise the health service and community awareness of the programme so that appropriate patients are identified and made aware of the supports available to them. As the programme has already been integrated into the standard practice of the health service, the health service should pursue more advertising and marketing as a part of the role of the coordinator of Connectors and the Connectors themselves playing a major role in communicating the programme benefits at the community level.

Pragmatically, at this stage in the programme’s development, the connector role needs to be promoted to health services in terms of enhancing surveillance and mitigating risk and to families as providing more options through enhanced social networks. The connector role looks different depending on whether it is viewed from within the service or within the family. Nevertheless, for Connectors to retain the public health character of their role, they need to be engaging with all domains of the new essentials model of comprehensive end-of-life care:^32^ that is, have constructive relationships with civic entities and informal networks, as well as with health services. Therefore, this connector role has been described as a distinct form of end-of-life volunteering,^11^ as it is accountable to a community rather than to a specific health service.

Nevertheless, families and referring healthcare professionals have from their differing perspectives strongly endorsed this model of care as feasible and acceptable, as did the Connectors in our previous publication.^27^ These evaluations together provide a solid basis to continue to develop the programme as a public health intervention and to embed within it robust continuous quality-improvement measures that will give further insight into the complexities of community engagement that connects formal and informal care systems.

### Strengths and limitations

Public health palliative care interventions, such as this community-based intervention, are implemented in real-world settings which are complex systems to undertake research, and so present a challenge to traditional research methodologies.^33^ This pilot programme was designed as a practical intervention, with an evaluation component about feasibility. The interviews were brief and focused on eliciting the two target groups’ overall perception of the benefits of the programme. They were not intended to provide data for an in-depth study of personal insights. The shortest interviews were repeat encounters in which health professionals, who had already been interviewed about one patient, had little or nothing to add to that report when followed up about another patient. While respondents were asked for suggestions as to how the programme might be improved, and a number of constructive comments were made, there was little negative response to the programme as a whole. The interviewer was not involved in the implementation of the programme to cause any biases.

The biggest strength of this study is that it led to a rapid translation into practice by the health service when managers reviewed the preliminary findings and were reassured by the feedback from their patients and staff that this initiative is feasible and acceptable to them and can be integrated into their standard practice.

## Conclusion

Evaluations comprising experiences of patients and service providers enable interventions to be translated into standard practice in healthcare and enable formal networks to work in partnership with informal community networks.

In this article, the perspectives of patients/families and healthcare providers demonstrated the mediating role of Connectors. Each group sees the Connectors’ contribution through the lens of their particular interests or needs. However, the effect of good connection is to shift the way care is understood and practised by each group, encouraging (or restoring) agency to families and reminding healthcare providers that respecting the boundaries of their roles and collaborating beyond those roles actually enhances the whole ecology of care.

Health systems would benefit from more coordinated and integrated care to better address what matters most to people, and certainly this study, like others, showed that these apparently small actions have an impact on health and need careful consideration. Using a Compassionate Communities–like approach to mobilising health and community sectors will lead to a more holistic approach that addresses not just the ‘big things’ but also these ‘little things’ in the social, practical and emotional domains, seemingly rarely touched by formal services.

## Declarations

**Ethics approval and consent to participate:** The study was conducted according to the guidelines of the Declaration of Helsinki and approved by the WACHS Human Research Ethics Committee (RGS3419) and La Trobe University Research Ethics Committee.

**Consent for publication:** Participants provided written informed consent before the start of data collection.

**Availability of data and materials:** Ethical approval precludes the data being used for another purpose or being provided to researchers who have not signed the appropriate confidentiality agreement. Specifically, the ethical approval specifies that all results are in aggregate form to maintain confidentiality and privacy and precludes individual-level data being made publicly available. All aggregate data for this study are freely available and included in the article. Interested and qualified researchers may send requests for additional data to Samar Aoun at samar.aoun@perron.uwa.edu.au
